# Postnatal counseling promotes early initiation and exclusive breastfeeding: a randomized controlled trial

**DOI:** 10.3389/fnut.2025.1473086

**Published:** 2025-02-28

**Authors:** Belda Negesa Beyene, Wako Golicha Wako, Dureti Moti, Alo Edin, Derese Eshetu Debela

**Affiliations:** ^1^Department of Midwifery, Institute of Health, Bule Hora University, Bule Hora, Oromia, Ethiopia; ^2^School of Public Health, Institute of Health, Bule Hora University, Bule Hora, Oromia, Ethiopia; ^3^Department of Epidemiology, School of Public Health, Institute of Health, Bule Hora University, Bule Hora, Oromia, Ethiopia; ^4^Department of Midwifery, College of Health and Medical Sciences, Madda Walabu University, Bale Robe, Oromia, Ethiopia

**Keywords:** breastfeeding, early initiation of breast feeding, exclusive breast feeding, immediate counseling, randomized controlled trial, postnatal women, Ethiopia

## Abstract

**Background:**

Breastfeeding plays a crucial role in promoting the health and wellbeing of both mothers and their infants, contributing to healthier populations and reducing long-term healthcare costs. Encouraging breastfeeding through education, support, and policies is essential for maximizing these benefits. The purpose of this study was to evaluate the impact of immediate postpartum counseling on early initiation of breastfeeding rates and exclusive breastfeeding in Bule Hora Teaching and Yabelo General Hospitals.

**Methods:**

A cluster randomized single-blinded trial was conducted on 224 postpartum women (112 in the intervention group and 112 in the control group) from January 1, 2023, to May 30, 2023. The effects of immediate postpartum counseling and its associated variables on early initiation and exclusive breastfeeding practices were evaluated using logistic regressions. A *p*-value of less than 0.05, a 95% confidence interval, and an adjusted odds ratio were used to determine statistical significance.

**Results:**

The proportion of early initiation of breastfeeding was significantly greater in the intervention group than in the control group (54.1% vs. 45.9%, *p* = 0.001), and exclusive breastfeeding practice were also significantly greater among women who received immediate breastfeeding counseling than among those who did not receive it (61.9% vs. 38.1%, *p* = 0.015). Those mothers who had an ANC visit for their pregnancy were three times more likely (AOR = 3.01, 95% CI = 1.12–8.1) to initiate breastfeeding. Having good knowledge regarding breastfeeding made them six times more likely to initiate breast-feeding earlier (AOR = 6.18, 95% CI = 1.77–21.57). Women who received counseling (AOR = 3.36, 95% CI: 1.83–6.16) and women who had good knowledge about breastfeeding (AOR = 1.88, 95% CI: 1.01–3.49) were significantly associated with exclusive breastfeeding practices.

**Conclusion:**

This study revealed that immediate postpartum breastfeeding counseling can positively influence both early initiations of breastfeeding and exclusive breastfeeding practices. Providing sustained education to women regarding early initiation and exclusive breastfeeding practices should be strengthened.

## Background

Exclusive breastfeeding means when the infant consumes only breast milk with no supplementation of other liquids or solids, not even water, with the exception of oral hydration solution or syrups of vitamins, minerals, or medicine (1, 2). The World Health Organization and the American Academy of Pediatrics recommend initiation of breastfeeding within an hour of birth, exclusive breastfeeding for the first 6 months, and continuing to breastfeed after solid foods are introduced as long as you and your baby desire, for 2 years and beyond (3, 4).

Despite the various advantages of breast milk for mothers (lowering the risk of breast and ovarian cancer, high blood pressure, and type 2 diabetes) and for babies (the best source of nutrition, protecting babies by lowering the risk of asthma, obesity, type 1 diabetes, and severe lower respiratory and gastrointestinal infection) (4, 5), the proportion of exclusive breastfeeding varies globally, which is 51.3% in Uttar Pradesh and 46.3% nationwide (6), 37% in sub-Saharan Africa (7). A South African survey revealed that 63.3% of participants initiated breastfeeding within the first hour, with 31.2% exclusively breastfeeding for the first 6 months and 58.97% in Ethiopia (8). Other evidence has shown that exclusive breastfeeding initiation rates range from 72 to 83% (9, 10).

A randomized controlled trial in urban Bangladesh reported a higher EBF rate (89.1%) in the intervention group than in the control group (77.4%) (11). Similarly, a rural Ethiopian cluster randomized controlled trial revealed that breastfeeding education and support significantly increased EBF by 25.9% (12).

Even though the World Health Organization recommends exclusive breastfeeding for approximately 6 months and continuing breastfeeding until at least 2 years of age, only approximately one-third of infants feed breast milk exclusively globally (2), and approximately 45% continue breastfeeding for at least 2 years, which is also low (13).

Nearly 96% of infant deaths within the first 6 months, representing 1.24 million deaths, are linked to nonexclusive breastfeeding. Furthermore, nonexclusive breastfeeding is associated with 55% of diarrhea deaths and 53% of acute respiratory deaths in infants under 6 months of age (7, 14). Suboptimal breastfeeding in Ethiopia contributes to 70,000 infant deaths annually, preventable through exclusive breastfeeding promotion. Effective interventions, such as the Baby Friendly Hospital Initiative, peer support, and community awareness campaigns are crucial to empower mothers and improve breastfeeding rates (7).

Several factors were found to influence early breastfeeding initiation. Older mothers (41–50 years), higher levels of education, greater gestational age, longer birth intervals, more prenatal care visits, and hospital deliveries were associated with a greater likelihood of initiating breastfeeding within the first hour (9, 10, 15).

Postpartum depression can make it difficult for mothers to breastfeed successfully. Mothers with depression often find it challenging to initiate and maintain breastfeeding because of fatigue, low mood, and difficulty bonding with their baby (16).

Receiving breastfeeding education and support from healthcare providers, possessing good knowledge about breastfeeding, having a positive attitude toward breastfeeding, being married, having secondary education or higher, accessing media, having 3–4 living children, and residing in rural areas are all significantly associated with the practice of exclusive breastfeeding (12, 17–19).

According to Global Breastfeeding Collective (UNICEF and WHO) reports, the rate of EBF in the first 6 months is 48% for 2023, which is close to the World Health Assembly target of 50% by 2025. The percentage of countries with at least 75% of care givers of children under 2 years of age counseled on infant and young child feeding in 2023 was 33%, with the 2030 target of the collective being 60% (20).

The findings of this study provide valuable data to inform potential interventions and support the collective 2030 target. Existing studies demonstrate a strong correlation between maternal education, knowledge, and delivery methods and exclusive breastfeeding (EBF) practices, the effectiveness of immediate postpartum counseling in promoting EBF remains significantly underexplored. Moreover, the geographical setting of the geographical setting of this area presents unique characteristics that may influence the applicability of those findings. Therefore, this study aimed to determine the impact of individualized immediate postpartum counseling on EBF and breastfeeding initiation among postpartum women in this specific geographical context.

## Materials and methods

### Study area and period

This study was conducted at Bule Hora University Teaching Hospital and Yabelo general hospitals in Oromia, Ethiopia, from January 1 to May 30, 2023. Bule Hora University Teaching Hospital is located in Bule Hora town, approximately 468 km south of Addis Ababa, the capital city, and serves as the capital of the West Guji Zone. Yabelo general hospital is situated in Yabelo town, approximately 570 km south of Addis Ababa, and serves as the capital of the Borena Zone. Both hospitals provide comprehensive healthcare services, including antenatal care, immunization, family planning, and delivery services, to their respective catchment areas.

### Study design

This study employed a cluster randomized controlled trial design to evaluate the effectiveness of individualized, immediate postnatal breastfeeding counseling on exclusive breastfeeding within the first month of life. The study clusters were defined by the number of weeks after childbirth. The appropriate number of weeks for the study was calculated, and random selections of these weeks were allocated to both groups. The remaining weeks were assigned to the control group. Women who delivered during the weeks assigned to the intervention group received the specific counseling intervention being tested, whereas those who delivered during the control group received only the standard postnatal care provided prior to the study. This ensured that the control group did not receive the intervention, allowing for a comparison of EBF rates between the two groups.

### Populations

#### Source population

The source population for this study was women who gave birth by spontaneous vaginal delivery at Bule Hora and Yabelo general hospitals.

#### Study population

All randomly selected women who gave birth by spontaneous vaginal delivery at Bule Hora and Yabelo hospitals during the study period.

### Eligibility criteria

Women who gave birth by spontaneous vaginal delivery at either Yabelo or Bule Hora Hospital during the data collection period or who were willing to participate in the study were included. Those women who were seriously ill and planned to move during the study period were excluded.

### Sample size determination and sampling procedure

Sample size for determining the number of clusters to be included in either the intervention or the control group. The sample size for the cluster randomized control trial was calculated on the basis of the following assumptions:

The average cluster size was 28 (i.e., on average, 28 eligible women were expected to give birth at Yabelo or/and Bule Hora Hospital, according to reports from the hospitals).

The prevalence of exclusive breastfeeding among newborns 0–1 months of age (i.e., primary outcome) in the control group was 74% (21) and the corresponding proportion in the intervention group was 94% (i.e., we expect a 20% improvement).

- Power of the study = 90%.
- Confidence level = 95%.

The coefficient of variations of the proportions is 1. The required sample size was subsequently calculated as follows:

$$c=1+\frac{{\left(Z_{1}+Z_{2}\right)}^{2}2p\left(1-p\right)/n+k^{2}\left(p_{1}^{2}+p_{2}^{2}\right)}{{\left(p_{2}-p_{1}\right)}^{2}}$$

where,

*c* = Number of clusters (i.e., “number of weeks”) required per group.

*Z*_1_ = Percentage point for *α* error.

*Z*_2_ = Percentage point for *β* error.

*p*_1_ = Proportion of primary outcome in the intervention group.

*p*_2_ = Proportion of primary outcome in the control group.

*p* = Average of p1 and p2.

*n* = Number of individuals in each cluster.

*k* = coefficients of variation of proportions among clusters in each group.

After the numbers were substituted into the formula, 3.375 clusters were required per group.

Finally, after rounding to a whole number, four clusters (i.e., weeks) per group were included in the study. Within each week, 28 eligible women are expected to give birth at both hospitals, in total, 2*4*28 = 224 women were included in this study.

During the study period, eight consecutive weeks were randomly allocated to either the intervention group or the control group. All eligible women within both the intervention and control groups were asked for consent and enrolled in the study. The information contamination was minimized by keeping the buffer period between the discharge of the first group and admissions of the second group. Additionally, random assignment of participants and blinding were used to minimize the contamination of information.

### Intervention

The intervention to be provided to the intervention group is package immediate postnatal counseling about exclusive breastfeeding provided at health facilities. All postpartum women enrolled in the intervention group received postnatal counseling in addition to usual postnatal care, using a counseling manual prepared for this study. The prepared manual was adapted from the World Health Organization guidelines for the counseling of women to improve breastfeeding practices (22). Four trained senior midwives (counselors) working at the labor unit of health facilities in the intervention group were trained on the package of immediate postnatal exclusive breastfeeding counseling and were supported in providing the counseling to women in the intervention group. Women in the control group received the usual package of postnatal care.

The counseling given for intervention was based on the individual mother’s circumstances, such as previous breastfeeding experiences, any complications during delivery, or specific concerns about feeding, which is more personalized by including detail information on breastfeeding, addressing different common challenges, offering emotional reassurance and encouragement to mothers and providing this support immediately after birth to establish more effective breastfeeding.

It is designed to cover several key topics related to breastfeeding. First and foremost, it emphasizes the importance of exclusive breastfeeding, including guidance on early initiation, the recommended duration, frequency, and techniques for proper positioning and attachment to ensure effective breastfeeding. The counselors also discuss the numerous advantages of EBF, highlighting how it helps prevent neonatal infections such as diarrhea, fever, cough, and pneumonia, while simultaneously reducing the risk of maternal diseases such as breast cancer and obesity.

Additionally, the counseling addresses the timing for introducing complementary foods and appropriate weaning options, ensuring that mothers are well_informed about the transition from exclusive breastfeeding. It also outlines the negative effects of nonexclusive breastfeeding, or mixed feeding, on both mothers and babies, including increased susceptibility to infectious diseases, potential retardation of fetal growth, decreased maternal–infant bonding, and risk of malnutrition. Finally, the counseling includes vital information on postpartum neonatal danger signs, such as fever, irritability, poor breastfeeding or inability to breastfeed, umbilical infections, and seizures. This comprehensive approach ensures that mothers receive critical information about breastfeeding practices and their implications for both their own health and that of their infants.

The control group received standard postnatal care, including routine check-ups and general breastfeeding advice. This advice encouraged frequent breastfeeding, both day and night, and emphasized the importance of exclusive breastfeeding for the first 6 months. The control group did not receive any specialized, individualized postnatal counseling. They served as a baseline for comparison with the intervention group.

### Outcomes

#### Primary outcome

The proportion of newborns who started early initiation of breastfeeding was a primary outcome.

#### Secondary outcomes

The effects of immediate breastfeeding counseling on breastfeeding frequency, maternal knowledge and maternal attitudes were secondary outcomes.

### Data collection tools and procedures

Data were collected by four trained midwives holding Bachelor of Science degrees. Two senior public health professionals supervised the overall data collection process. The questionnaire was initially prepared in English, then translated into both Afaan Oromo and Amharic, and subsequently back-translated into English to ensure accuracy. Both the data collectors and supervisors received a one-day training.

A pre-tested, structured and adapted questionnaire (23, 24) was used to collect the data. Face-to-face interviews were conducted at the women’s homes, after the data collectors located the homes by telephone.

Data collection occurred in two stages. In the first stage, midwives at the labor ward obtained informed consent from eligible women and collected preliminary data, including address information, to enable prospective data collectors to trace the women’s homes within the communities. This stage involved collecting address data from eligible women discharged from hospitals. In the second stage, trained data collectors contacted these women at their homes on the 30th day postpartum and collected the required end-line data.

### Study variables

#### Dependent variable

Early breastfeeding initiation was the primary outcome; breastfeeding frequency, maternal knowledge, and attitudes were secondary outcomes.

#### Independent variables

Sociodemographic factors: maternal age, marital status, maternal education, maternal occupation, and monthly family income.

Obstetric factors: Parity, antenatal care, postnatal counseling about EBF, breastfeeding counseling, place and mode of delivery, HIV status of mothers, colostrum feeding, and timely initiation of breastfeeding.

### Operational definitions

Control group: Women who did not receive immediate individualized postnatal counseling.

Intervention group: Women who received immediate individualized postnatal counseling.

Exclusive breastfeeding: defined as an infant consuming only human milk without any other liquid supplements (23).

Early initiation of breastfeeding: refers to the practice of a mother starting to provide breast milk to her newborn within the first hour after birth (10).

Prelacteal feeding: is defined as the practice of giving a newborn something to eat or drink (water, sugar water, formula, or other liquids) before they are breastfed for the first time.

Immediate postpartum breastfeeding counseling: refers to counseling mothers about breastfeeding during the first 24 h following childbirth (25).

Frequency of breastfeeding: This study investigated how often mothers breastfed their babies. They considered a mother to be “frequently breastfeeding” if she fed her baby on demand, which meant breastfeeding 8–12 times per day. They assigned a “1” to mothers who breastfed on demand and a “0” to mothers who breastfed less often (fewer than 8 times a day).

Attitude: In this study, 12 items are used to assess respondents’ attitudes measured on a five-point Likert scale, where respondents can express their level of agreement from “strongly agree” to “strongly disagree.” Each response is subsequently transformed into a binary scale for analysis: a score of “1” is assigned for agreement with positively framed questions, whereas a score of “0” is given for disagreement. Conversely, for negatively framed questions, the score is reversed “1” for disagreement and “0” for agreement. When an individual’s overall score equals or exceeds the mean score of the breastfeeding-related items, it is considered that they have a positive attitude toward breastfeeding. In contrast, scores falling below the mean indicate a negative attitude toward breastfeeding. This method allows for a clear categorization of attitudes on the basis of the respondents’ answers (26).

Knowledge: Mothers’ breastfeeding knowledge was assessed using an eight-item questionnaire. Scores at or above the mean were considered good knowledge; scores below the mean were considered poor knowledge (26).

Wealth Index: Household wealth status was determined using a five-quintile wealth index (poorest, poorer, middle, rich, richest). The poorest individuals were labeled ‘1’, the poorer individuals were labeled ‘2’, the middle quintile ‘3’, the rich quintile ‘4’, and the richest quintile ‘5’ (27, 28).

### Data quality control

The data collection questionnaire was revised to be complete and appropriate before the commencement of data collection. Possible corrections were made at the end of every day of data collection to check if there were any unnecessary or missing variables before accepting the data and starting to use it. To determine the clarity and understandability of the data collection instrument, a pretest was conducted on 22 women (10% of the sample size) from Adola town who gave birth at Adola General Hospital.

### Data processing and analysis

The collected data were checked for completeness, coded, and entered into EPI Data Version 4.6. Data analysis was conducted via SPSS version 25. Descriptive statistics, including means, standard deviations, percentages, and frequencies, were calculated. Pearson’s chi-square test was used to assess differences in sociodemographic and obstetric variables, as well as to compare the frequency of breastfeeding, knowledge, and attitudes between the intervention and control groups. Principal component analysis was used to calculate the household wealth index.

Model goodness of fit was confirmed (*p*-value = 0.56), and multicollinearity tests for EIBF and EBF revealed no collinearity among the independent variables (values of 0.65 and 0.72, respectively). The effects of counseling interventions on primary and secondary outcomes are expressed using adjusted odds ratios (AORs) and *p*-values, with statistical significance declared at *p* < 0.05. The results are presented in text, tables, and graphs.

## Results

### Sociodemographic and economic characteristics of the mothers

**Figure 1:**
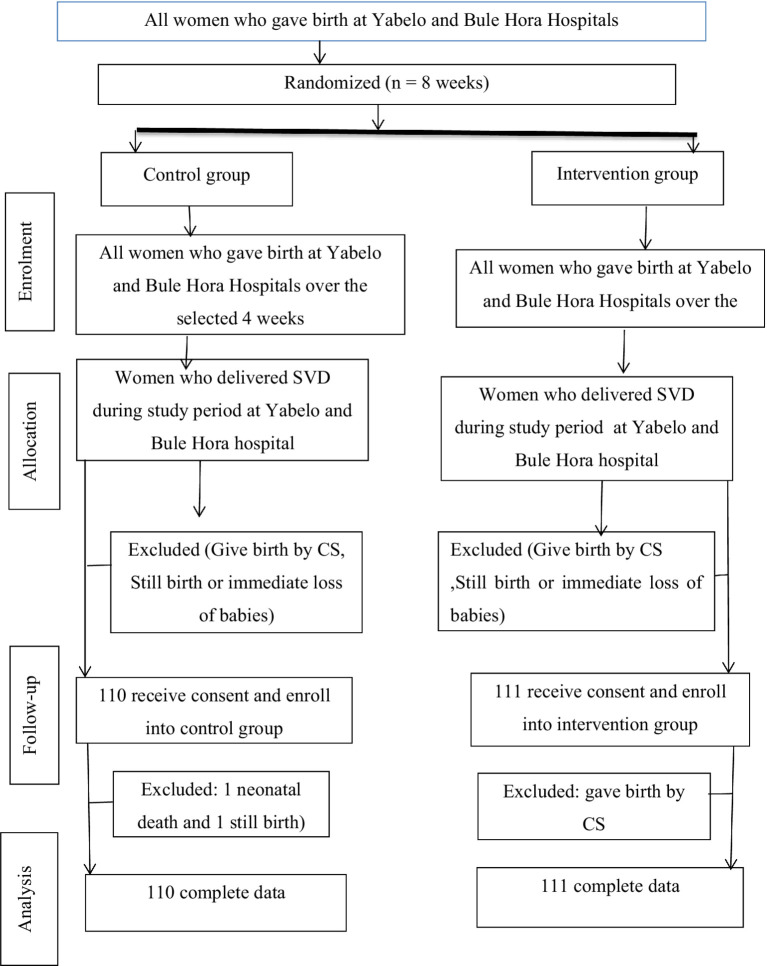
Consolidated standards of reporting trials flow diagram of the study.

About 224 (112 treatment and 112 control) postnatal women were included at the start of the study. Among these samples, 110 and 111 from the control and treatment groups, respectively, were included. At the one-month postpartum follow-up, 3 subjects (n intervention = 1; control = 2) were excluded. The reasons for dropout were reported according to the CONSORT reporting guidelines (Figure 1). The mean age (±SD) of the control and treatment groups was 24.35 (±4.16) and 24.73 (±4.26) years, respectively (Table 1).

**Table 1:** Baseline characteristics of mothers in hospitals in Oromia, Ethiopia.

Variables	Category	Control (*N* = 110)	Treatment (*N* = 111)	*p*-value
Study site	Yabelo	51 (51)	49 (49)	0.74
	Bule Hora	59 (48.8)	62 (51.2)	
Age	less than or equal to 24	56 (50)	56 (50)	0.88
	25–29	39 (50.6)	38 (49.4)	
	30–34	13 (50)	13 (50)	
	greater than or equal to 35	2 (33.3)	4 (66.7)	
Marital status	Married	107 (50)	107 (50)	0.61
	Divorced	2 (50)	2 (50)	
	Widowed	1 (33.3)	2 (66.7)	
Religion	Orthodox	17 (40.5)	25 (59.5)	0.56
	Protestant	65 (50.8)	63 (49.2)	
	Muslim	27 (55.1)	22 (44.9)	
	Waqefata	1 (50)	1 (50)	
Educational status	No formal education	4 (36.4)	7 (63.6)	0.45
	Primary (grade 1-8)	35 (50)	35 (50)	
	Secondary (grade 9-12)	30 (44.8)	37 (55.2)	
	College and above	41 (56.2)	32 (43.8)	
Occupation	Government employee	32 (49.2)	33 (50.8)	0.89
	Private	5 (41.7)	7 (58.3)	
	Daily laborer	10 (52.6)	9 (47.4)	
	House wife	56 (51.9)	52 (48.1)	
	Merchant	7 (41.2)	10 (58.8)	
Wealth index	Poorest	23 (50)	23 (50)	0.28
	Poor	15 (38.5)	24 (61.5)	
	Rich	72 (52.9)	64 (47.1)	

### Obstetric characteristics of the respondents

In this study, about 64 (48.9%) women in the control and 67 (51.1%) in the intervention were multipara. Half (50 %) of both the intervention and control groups had antenatal care attendance (Table 2).

**Table 2:** Obstetric characteristics of mothers in hospitals in Oromia, Ethiopia.

Variables	Category	Control (*N* = 110)	Treatment (*N* = 111)	*p*-value
Parity	Primiparous	36 (53.7)	31 (46.3)	0.66
	Multipara	64 (48.9)	67 (51.1)	
	Grand multipara	10 (43.5)	13 (56.5)	
ANC follow-up	Yes	96 (50)	96 (50)	0.86
	No	14 (48.3)	15 (51.7)	
Frequency of ANC visits	1	3 (75)	1 (25)	0.31
	2–3	41 (45.1)	50 (54.9)	
	4 and above	62 (53)	55 (47)	
Newborns sex	Male	54 (51.9)	50 (48.1)	0.55
	Female	56 (47.9)	61 (52.1)	
Birth order	1	35 (53)	31 (47)	0.74
	2–3	55 (49.5)	56 (50.5)	
	4 and above	20 (45.5)	24 (54.5)	

### Mothers’ knowledge characteristics about breastfeeding

There was no significant difference in antenatal breastfeeding counseling between the intervention (52.3%) and control groups (47.7%) (*p* = 0.08). However, a significantly greater proportion of respondents in the control group (54.5%) than in the intervention group (45.5%) recognized the benefit of EBF for baby growth (*p* = 0.03) (Table 3).

**Table 3:** Knowledge characteristics of mothers in hospitals in Oromia, Ethiopia.

Variables	Category	Control (*N* = 110)	Treatment (*N* = 111)	*p*-value
Receive counseling during ANC	No	94 (47.7)	103 (52.3)	0.08
	Yes	16 (66.7)	8 (33.3)	
Receive counseling during childbirth	No	60 (52.2)	55 (47.8)	0.46
	Yes	50 (47.2)	56 (52.8)	
Receive counseling during PNC	No	42 (53.8)	36 (46.2)	0.37
	Yes	68 (47.6)	75 (52.4)	
Advantages of EBF for newborn				
Good food for baby	No	58 (53.2)	51 (46.8)	0.31
	Yes	52 (46.4)	60 (53.6)	
Prevents disease	No	93 (51.4)	88 (48.6)	0.30
	Yes	17 (42.5)	23 (57.5)	
Bond mother and baby	No	94 (51.1)	90 (48.9)	0.38
	Yes	16 (43.2)	21 (56.8)	
Good for baby growth	No	25 (38.5)	40 (61.5)	0.03
	Yes	85 (54.5)	71 (45.5)	
Advantages of EBF for mother				
Help in recovery of the uterus	No	94 (47.7)	103 (52.3)	0.08
	Yes	16 (66.7)	8 (33.3)	
Prevents another pregnancy	No	60 (52.2)	55 (47.8)	0.37
	Yes	50 (47.2)	56 (52.8)	
Save money	No	42 (53.8)	36 (46.2)	0.99
	Yes	68 (47.6)	75 (52.4)	
Bond mothers and baby	No	108 (49.8)	109 (50.2)	0.67
	Yes	2 (50)	2 (50)	
Prevent disease	No	42 (38.2)	36 (32.4)	0.37
	Yes	68 (61.8)	75 (67.6)	
Overall knowledge status	Poor	69 (56.6)	53 (43.4)	0.014
	Good	41 (41.4)	58 (58.6)	

### Mothers’ attitudes toward breastfeeding

Nearly, both the intervention and treatment groups thought that breastfeeding improved the bond between the mother and the baby. About 67 (57.3%) and 50 (42.7%) of the intervention and control groups, respectively, had positive attitudes (Table 4).

**Table 4:** Mothers’ attitudes toward breastfeeding in hospitals in Oromia, Ethiopia.

Variables	Category	Control (*N* = 110)	Treatment (*N* = 111)	*p*-value
After baby start using additional food or fluid breast milk is useless	Agree	26 (65)	14 (35)	0.03
	Disagree	84 (46.4)	97 (53.6)	
Breastfeeding is not good because it negatively affect the mother shape	Agree	5 (62.5)	3 (37.5)	0.46
	Disagree	105 (49.3)	108 (50.7)	
Breastfeeding improve bond between mother and baby	Disagree	2 (66.7)	1 (33.3)	0.32
	Agree	108 (49.5)	110 (50.5)	
When mothers wants to workout do formula milk is better than breast milk	Agree	69 (51.5)	65 (48.5)	0.44
	Disagree	40 (46.5)	46 (53.5)	
Mother who formula feed baby lost tone criterium for motherhood	Agree	100 (53.5)	87 (46.5)	0.01
	Disagree	10 (29.4)	24 (70.6)	
Breast feed baby is healthier than formula feed baby	Disagree	2 (33.3)	4 (66.7)	0.41
	Agree	108 (50.2)	107 (49.8)	
Breast milk is ideal food for baby	Disagree	3 (50)	3 (50)	0.99
	Agree	107 (49.8)	108 (50.2)	
Breast milk is better than formula milk	Disagree	1 (25)	3 (75)	0.08
	Agree	109 (50.2)	108 (49.8)	
Breast milk is cheaper than formula milk	Disagree	83 (48.5)	88 (51.5)	0.45
	Agree	27 (54)	23 (46)	
Breast milk is more digestible than formula milk	Disagree	1 (50)	1 (50)	0.99
	Agree	109 (49.8)	110 (50.2)	
Overall attitude status	Negative	60 (57.7)	44 (42.3)	0.026
	Positive	50 (42.7)	67 (57.3)	

### Practice of participants toward breastfeeding

The majority of participants in both the control and treatment groups reported providing prelacteal feeding (48.8% vs. 51.2%), indicating a common practice. However, there was a significant difference in breastfeeding initiation, with the control group showing a higher prevalence of delaying breastfeeding beyond 1 h after delivery (80% vs. 20%).

Interestingly, colostrum feeding was rare in both groups, with only a small percentage of participants reporting feeding colostrum to their newborns.

Regarding additional fluids, both groups reported a low prevalence of providing fluids other than breast milk to their newborns. Similarly, the introduction of solid or semisolid food was infrequent in both groups. A significantly greater proportion of babies in the intervention group (53%) received frequent breastfeeding, as per the infant’s needs, compared to the control group (47%) (*p* = 0.016) (Table 5).

**Table 5:** Practice of participants toward breastfeeding in hospitals in Oromia, Ethiopia.

Variables	Category	Control, *N* (%)	Treatment, *N* (%)	*p*-value
Presence of prelacteal feeding	Yes	105 (48.8)	110 (51.2)	0.096
	No	5 (83.3)	1 (16.7)	
After how many hours after delivery start breast feeding	Within 1 h	90 (45.9)	106 (54.1)	0.001
	After 1 h	20 (80)	5 (20)	
Did you feed colostrum to the newborn	Yes	1 (50)	1 (50)	0.99
	No	109 (49.8)	110 (50.2)	
Did you ever feed any fluid except breast milk to the newborn	Yes	4 (80)	1 (20)	0.17
	No	106 (49.1)	110 (50.9)	
Did you provide solid or semi solid food to newborn	Yes	1 (33.3)	2 (66.7)	0.57
	No	109 (50)	109 (50)	
How frequent baby should get breast milk in a day	On demand (8–12 times per day)	93 (47)	105 (53)	0.016
	Not on demand (<8 times per day)	17 (74)	6 (26)	
	Baby should get breast milk during illness	No	7 (50)		7 (50)	0.98
Yes		103 (49.8)	104 (50.2)			

### Proportion of newborns who start early breastfeeding

About 106 (54.1%) of the neonates in the intervention group and 90 (45.9%) of those in the control group breastfed started early initiation of breastfeeding, respectively (*p* = 0.001).

### Factors associated with early initiation of breastfeeding

This study investigated the associations between various factors and early breastfeeding initiation. Bivariate analysis revealed significant associations (*p* < 0.25) between early breastfeeding initiation and maternal education, occupation, antenatal care, knowledge status, childbirth counseling, and postnatal care. Multivariable analysis adjusted for confounding factors, revealed a significant and independent association between early breastfeeding initiation and both antenatal care follow-up and good breastfeeding knowledge. Compared with those who did not receive antenatal care, women who received antenatal care were three times more likely to initiate breastfeeding early (AOR = 3.01, 95% CI = 1.12–8.1). Similarly, mothers with good breastfeeding knowledge were six times more likely to initiate breastfeeding early (AOR = 6.18, 95% CI = 1.77–21.57) than their counterparts were (Table 6).

**Table 6:** Factors associated with EIBF in hospitals, Oromia, Ethiopia (*N* = 221).

Variables	Category	EIBF		COR (95%Cl)	AOR (95%Cl)
		Yes, *N* (%)	No, *N* (%)		
Mother occupation	Gov’t employee	56 (86.2)	9 (13.8)	0.39 (0.046–3.39)	0.58 (0.06–6.01)
	Private employee	9 (75)	3 (25)	0.188 (0.017–2.07)*	0.79 (0.05–11.3)
	Daily laborer	18 (94.7)	1 (5.3)	1.12 (0.065–19.49)	7.13 (0.27–18)
	House wife	97 (89.8)	11 (10.2)	0.55 (0.067–4.57)	1.21 (0.13–11.52)
	Merchant	16 (94.1)	1 (5.9)	1	1
Mother education	No schooling	8 (72.7)	3 (27.3)	1	1
	Primary education	65 (92.9)	5 (7.1)	4.87 (0.98–24.36)*	5.51 (0.96–31.77)
	Secondary education	58 (86.6)	9 (13.4)	2.42 (0.54–10.84)*	2.36 (0.45–12.36)
	College and above	65 (89)	8 (11)	3.05 (0.67–13.88)	2.69 (0.51–14.22)
ANC follow-up	Yes	175 (91.1)	17 (8.9)	3.92 (1.51–10.18)	3.01 (1.12–8.10)**
	No	21 (72.4)	8 (27.6)	1	1
Counseling during childbirth	Yes	98 (92.5)	8 (7.5)	2.12 (0.88–5.15)	1.07 (0.34–3.33)
	No	17 (14.8)	98 (85.2)	1	1
Counseling during PNC	Yes	130 (90.9)	13 (9.1)	1.82 (0.79–4.21)	1.59 (0.61–4.14)
	No	66 (84.6)	12 (15.4)	1	
Knowledge status	Good	96 (97)	3 (3)	7.04 (2.04–24.29)	6.18 (1.77–21.57)**
	Poor	100 (82)	22 (18)	1	1

*Significant at *p* value <0.25, **significant at *p* value <0.05, AOR: adjusted odds ratio; COR: crude odds ratio; 1: reference.

### Effects breastfeeding on frequency, knowledge and attitude

The intervention group showed a significantly higher proportion of newborns (53%) receiving frequent breastfeeding compared to the control group (47%) (*p* = 0.014). Immediate postnatal counseling significantly increased knowledge about breastfeeding by 17.2% (58.6% in the intervention group vs. 41.4% in the control group, *p* = 0.025). Additionally, the intervention group had a significantly greater proportion of mothers (57.3%) with a positive attitudes toward breastfeeding than did the control group (42.7%) (*p* = 0.026) (Table 7).

**Table 7:** The effects of breastfeeding counseling on frequency, knowledge, and attitudes among women in Oromia hospitals, Ethiopia.

Outcomes	Category	Treatment, *N* (%)	Control, *N* (%)	Pearson chi-square value
Frequency	Yes	105 (53)	93 (47)	0.014
	No	6 (26.1)	17 (73.9)	
Knowledge	Good	58 (58.6)	41 (41.4)	0.025
	Poor	53 (43.4)	69 (56.6)	
Attitude	Positive	67 (57.3)	50 (42.7)	0.026
	Negative	44 (42.3)	60 (57.7)	

### Factors associated with exclusive breastfeeding

Mothers’ educational status, presence of prelacteal feeding, knowledge status, attitude status, receiving counseling and receiving breastfeeding frequently were associated with bivariable logistic regression, with *p*-value less than 0.25. According to the multivariable analysis, receiving a counseling intervention, having good knowledge of breast feeding and having frequently feed breast feeding were significantly associated with exclusive breastfeeding practices. Women who received counseling intervention group were 3.36 times more likely (AOR = 3.36, 95% CI 1.83–6.16) to practice exclusive breastfeeding than those who did not gain. Additionally, women who had good knowledge about breastfeeding were nearly 2 times more likely (AOR = 1.88, 95% CI = 1.01–3.49) to practice exclusive breastfeeding than to their counterparts were (Table 8).

**Table 8:** Factors associated with EBF in hospitals in Oromia, Ethiopia (*n* = 221).

Variables	Category	EBF		OR (95%Cl)	AOR (95%Cl)
		Treatment, *N* (%)	Control, *N* (%)		
Mother education	No schooling	7 (63.6)	4 (36.4)	1	1
	Primary education	35 (50)	35 (50)	0.57 (0.15–2.13)	0.34 (0.08–1.44)
	Secondary education	37 (55.2)	30 (44.8)	0.70 (0.19–2.64)	0.35 (0.08–1.50)
	College and above	32 (43.8)	41 (56.2)	0.45 (0.12–1.66)*	0.21 (0.05–0.93)
Given prelacteal feeding	Yes	110 (51.2)	105 (48.8)	1	
	No	1 (16.7)	5 (83.3)	0.19 (0.02–1.66)	0.17 (0.02–1.57)
Received counseling	Yes	83 (61.9)	51 (38.1)	0.29 (0.16–0.51)	3.36 (1.83–6.16)**
	No	28 (32.2)	59 (67.8)	1	1
Knowledge	Good	58 (58.6)	41 (41.4)	0.54 (0.31–0.93)	1.88 (1.01–3.49)**
	Poor	53 (43.4)	69 (56.6)	1	1
Attitude	Positive	67 (57.3)	50 (42.7)	0.55 (0.32–0.93)	1.64
	Negative	44 (42.3)	60 (57.7)	1	1
BF Frequency	On demand (8–12 times per day)	105 (53)	93 (47)	0.31 (0.12–0.83)	5.93 (2.06–17.01)**
	Not on demand (<8 times per day)	6 (26.1)	17 (73.9)	1	

*Significant at *p* value <0.25, **significant at *p* value <0.05, AOR: adjusted odds ratio; BF: breastfeeding; COR: crude odds ratio; 1: reference.

## Discussion

To improve breastfeeding results and improve the wellbeing of both mothers and babies, breastfeeding counseling is an essential part of support. Its importance extends beyond simply teaching techniques to a holistic approach that empowers mothers and optimizes health benefits.

Hence, this study demonstrates the positive impact of immediate postpartum counseling on exclusive breastfeeding and frequent breastfeeding practices among mothers who deliver in hospitals.

The study revealed a significant association between early breastfeeding initiation and antenatal care attendance and good breastfeeding knowledge. Furthermore, frequent breastfeeding, counseling, and positive knowledge about breastfeeding were identified as key determinants of EBF practices.

The study also revealed a statistically significant association between breastfeeding frequency and both knowledge and attitudes toward breastfeeding. Compared with the control group, the intervention group presented a greater frequency of breastfeeding, improved knowledge about breastfeeding, and a more positive attitude toward breastfeeding. These findings suggest a positive correlation between frequent breastfeeding and both knowledge and attitudes regarding breastfeeding practices among women.

This study revealed that women receiving counseling intervention initiated breastfeeding significantly earlier than those in the control group did (54.1% vs. 45.9%). This aligns with findings from a cluster randomized controlled trial conducted in Bangladesh (81.9% vs. 77.4%) (11) and southern Ethiopia (23) (72.7%) vs. 59.9%, which also reported significantly higher rates of early breastfeeding initiation in the intervention groups. The difference in effect size might be due to a different population and a larger sample size in the previous study.

Research in Uttar Pradesh, India, indicated that 48.1% of mothers who received both prenatal and postnatal counseling initiated breastfeeding within the first hour (29), highlighting the positive impact of comprehensive support. These results highlight the value of counseling services to encourage the timely commencement of breastfeeding.

This could be explained by the education intervention, which could increase their drive to start breastfeeding at an early age and increase their understanding of the benefits of doing so. The breastfeeding education participants were 1.55 times more likely to start breastfeeding at an early age, according to a cluster randomized study done in southern Ethiopia (23).

In this study, having antenatal follow-up during their pregnancy was three times more likely to initiate early initiation of breastfeeding than was their counterpart. This finding is consistent with the studies conducted in both India (30) and Ethiopia (10), which revealed that those who have a greater number of antenatal care visits were 1.26 times more likely to start early breast feeding. These findings are also supported by studies conducted in South Gondor, Ethiopia (31). This may be due to the different awareness creation messages they received during their follow-up.

Having good knowledge was significantly (6.18 times) associated with an early start of breastfeeding. This finding agrees with a study investigated in Moshi Municipality, northern Tanzania (9), which concluded that those who were aware of timely BF initiation during pregnancy were three times more likely to initiate early breast feeding. This might be because good breastfeeding knowledge empowers mothers to initiate breastfeeding early by providing motivation, confidence, and awareness of benefits and risks. Successful early breastfeeding, in turn, strengthens mothers’ desire to learn more, increasing their confidence and promoting a successful breastfeeding journey.

Women who received counseling intervention also practiced exclusive breastfeeding at much higher rates than those who did not (61.9% vs. 38.1%), which agrees with a RCT conducted in northern India (85.5% vs. 64%) (32) and southern Ethiopia (74.1% vs. 60.6%) (23). This may be because women who have attended breastfeeding counseling are more prepared to comprehend the benefits of exclusive breastfeeding. Compared with mothers who did not receive education intervention, study participants who did receive it were 3.36 times more likely to exclusively breastfeed. This result is in line with a South Ethiopian study that reported exclusive breastfeeding was 1.72 times more common among women were in an education intervention group than among those in the control group (23).

However, studies conducted in Arabia have shown that there is no impact of counseling on exclusive breastfeeding (33). The sociodemographic characteristics from the prior study, which revealed that 96.2% of mothers had an education, may be responsible for this.

In the present study, mothers who had good knowledge of EBF were more likely to practice EBF compared to those who had less knowledge of EBF, which aligns with the findings of a study conducted in Ghana (34), which showed that those women who had greater knowledge of EBF were 5.9 times more likely to practice of EBF. This findings also agrees with the findings of study conducted in Lagos State, Nigeria, which indicated that respondents with good knowledge of EBF were three times more likely to practice EBF well than those with poor knowledge (18). This might be due to the need to educate mothers about EBF’s benefits, proper techniques, and potential challenges empowering them to make informed decisions and practice exclusive breastfeeding successfully. Therefore, promoting breastfeeding knowledge is essential for encouraging EBF.

Those who frequently breastfeed newborns are nearly six times more likely to practice EBF than their counterparts are, as supported by a study conducted in China (35), which revealed that those who frequently breastfeed were more likely to practice EBF. This is attributed to the fact that frequent breastfeeding stimulates milk production, ensuring a sufficient supply to meet the baby’s growing needs and supporting healthy weight gain. Furthermore, frequent breastfeeding fosters close physical and emotional bonds between mothers and children, strengthening their connection and creating a sense of security. Therefore, encouraging frequent breastfeeding is essential for promoting successful EBF and ensuring the optimal health and wellbeing of both mothers and babies.

## Strengths and limitations

Clustered randomized controlled trials offer a potential solution for limiting bias, particularly selection bias and confounding, and enabling blinding. This design ensures equal chances of participation, although it has with limitations. The generalizability of the study must be carefully considered. Maternal intrinsic motivation, which might influence the results, was not assessed.

## Conclusion and recommendations

The findings strongly suggest that individualized, immediate postnatal counseling on exclusive breastfeeding during the first month of life is an effective intervention for improving EIBF and EBF practices. This approach, combined with mothers having a history of ANC visits and good breastfeeding knowledge, significantly influenced early initiation of breastfeeding. Furthermore, this study highlights the crucial role of counseling in promoting good breastfeeding knowledge and frequent breastfeeding, which are key determinants of successful exclusive breastfeeding practices.

A comprehensive program that provides individualized, immediate postnatal counseling on EBF to all mothers within the first month of life should be implemented. This counseling should address individual needs and concerns, provide practical support, and reinforce the benefits of EBF.

Pregnant women were encouraged to attend ANC visits regularly. ANC visits should include comprehensive breastfeeding education, ensuring that mothers have a strong foundation of knowledge before giving birth.

Breastfeeding counseling should be integrated into existing maternal and child health programs. This ensures consistent access to information and support throughout the postpartum period.

Investing in training healthcare professionals, particularly midwives and nurses, to provide effective breastfeeding counseling and support. They are equipped them with the knowledge and skills to address individual needs and empower mothers to practice EBF successfully.

## Data Availability

The original contributions presented in the study are included in the article/Supplementary material, further inquiries can be directed to the corresponding author.
