# Supplementary material for: Postnatal counseling promotes early initiation and exclusive breastfeeding: a randomized controlled trial
Source: Front Nutr. 2025 Feb 28;12:1473086. doi: 10.3389/fnut.2025.1473086 (PMC11906318; doi:10.3389/fnut.2025.1473086)
Supplement: Supplementary file 1 [file Table_1.DOCX]

ANNEX III: STRUCTURED COUNSELING FORMAT

The structured counseling format to be given for the intervention group. Points on which counseling will be given:

**⮚ Awareness on exclusive breast feeding.**

✓ Early initiation of breast feeding.

✓ Duration of EBF.

✓ Frequency of breast feeding.

✓ Positioning and attachment for effective breast feeding.

⮚ Advantages of EBF and benefits of breast milk

✓ Neonatal infections prevented by appropriate breast feeding like diarrhea, fever, cough pneumonia.

✓ Maternal diseases prevented by breast feeding like breast cancer, overweight and obesity.

✓ Role of breast feeding in supporting uterine involution.

**⮚ About complementary breast feeding.**

✓ Time of starting

✓ Winning food items

⮚ **Effect of non-exclusive breast feeding (mixed breast feeding) on fetus and mother.**

✓ Susceptibility to infectious disease.

✓ Retardation of fetal growth.

✓ Decreased fetal maternal bonding.

✓ Malnutrition.

**⮚ About post-partum neonatal danger signs**

✓ Fever

✓ Irritability

✓ Poor breast feeding or unable to breast feed.

✓ Umbilical infection

✓ Seizure……etc.
